# Why Self‐Leadership Alone Is Not Enough: How Professional Self‐Concept and Organizational Commitment Shape Male Nurses’ Intention to Stay

**DOI:** 10.1155/jonm/3597841

**Published:** 2026-04-20

**Authors:** Donghyeok Lee, Mihwa Shim, Jungmin Lee

**Affiliations:** ^1^ Department of Nursing Education, Hallym University, Chuncheon, Gangwon-do, South Korea, hallym.ac.kr; ^2^ Nursing Department, Kangnam Sacred Heart Hospital, Hallym University, Seoul, South Korea, hallym.or.kr; ^3^ School of Nursing, Hallym University, Chuncheon, Gangwon-do, South Korea, hallym.ac.kr

**Keywords:** intention, leadership, nurses, organizational commitment, professional competence

## Abstract

**Background:**

Self‐leadership, conceptualized as self‐regulatory cognitive and behavioral strategies through which individuals influence their motivation and performance, has gained increasing attention in organizational research. However, its conceptual role in nursing—particularly in relation to retention among male nurses—remains insufficiently clarified. This study examined the association between self‐leadership and intention to stay among male nurses, alongside testing the mediating roles of professional self‐concept and organizational commitment.

**Methods:**

A cross‐sectional survey was conducted with 194 male nurses employed in general and tertiary hospitals in South Korea. Data were collected using validated self‐report instruments measuring self‐leadership, professional self‐concept, organizational commitment, and intention to stay. Parallel mediation analysis was performed using PROCESS macro with 10,000 bootstrap resamples to estimate indirect effects.

**Results:**

Self‐leadership was positively associated with professional self‐concept, organizational commitment, and intention to stay. When both mediators were included in the model, the direct effect of self‐leadership on intention to stay became nonsignificant, while significant indirect effects were observed through professional self‐concept and organizational commitment. These findings indicate that self‐leadership influences retention primarily through identity‐related and organizational attachment mechanisms.

**Conclusions:**

Self‐leadership appears to function as an upstream regulatory capacity that contributes to retention indirectly by strengthening professional identity and organizational commitment, rather than exerting a direct effect on retention decisions. These findings clarify the conceptual positioning of self‐leadership in nursing retention research and highlight the need for multilevel retention strategies.

**Implications for Nursing Management:**

Retention strategies for male nurses should integrate structured self‐leadership development with organizational initiatives that promote professional identity consolidation and inclusive workplace environments.

## 1. Introduction

Nurse retention has become a persistent and global challenge for health care organizations, as high turnover rates undermine workforce stability, patient safety, and organizational effectiveness [1–3]. Sustained nurse shortages are associated with increased workload, burnout, and compromised quality of care, ultimately threatening the sustainability of healthcare systems [4]. In response, healthcare organizations have increasingly shifted their focus from reducing turnover intention to strengthening intention to stay, which is considered a more proactive and stable indicator of workforce retention [5, 6].

Within this context, male nurses have gained attention as a potential workforce resource to alleviate nurse shortages and promote diversity in nursing organizations. Although the proportion of male nurses has gradually increased in many countries, their retention remains fragile compared with female nurses [7, 8]. Male nurses are more likely to experience early turnover and demonstrate lower intention to stay, highlighting structural vulnerabilities in their organizational integration [9, 10].

Nursing has historically evolved as a female‐dominated profession, shaped by gendered norms that associate caring roles with femininity [11, 12]. Despite increasing professionalization and technological advancement in healthcare, these gendered assumptions continue to influence organizational cultures and interpersonal dynamics within nursing workplaces [13]. Male nurses often occupy a minority status and encounter role incongruity, stereotyping, and marginalization during their professional socialization [14, 15]. Such experiences may weaken their sense of professional legitimacy and belonging, thereby influencing organizational commitment and retention decisions.

Retention is not solely determined by external working conditions but is also shaped by individual psychological resources and professional identity [16]. Among these resources, self‐leadership has been identified as a personal self‐regulatory capacity that enables individuals to influence their motivation, cognition, and behavior toward goal attainment through cognitive and behavioral strategies [17, 18]. Originally conceptualized by Manz, self‐leadership refers to a comprehensive process through which individuals direct and motivate themselves by applying specific behavioral and cognitive strategies, including self‐goal setting, self‐observation, self‐reward, constructive thought pattern strategies, and natural reward strategies. These strategies enhance intrinsic motivation and foster a sense of personal agency and responsibility in one’s professional role. Unlike traditional leadership, which focuses on influencing others, self‐leadership emphasizes internal self‐influence and autonomous regulation, making it particularly relevant in complex and dynamic healthcare environments where nurses are required to make independent clinical judgments and manage multifaceted responsibilities. In such contexts, self‐leadership may function as an internal resource that strengthens resilience, adaptive coping, and professional growth [18]. In nursing contexts, self‐leadership has been associated with job satisfaction, work engagement, organizational effectiveness, and reduced burnout [19]. However, evidence regarding its direct effect on retention‐related outcomes remains inconsistent, particularly with respect to intention to stay as a distinct and forward‐looking retention construct.

Some studies suggest that self‐leadership directly reduces turnover intention and enhances intention to stay [20], whereas others indicate that its influence may be indirect, operating through psychological and organizational mechanisms, such as professional identity, empowerment, or organizational commitment [16, 20]. These divergent findings suggest that self‐leadership may not function as a proximal determinant of retention but rather as an upstream regulatory resource whose impact depends on identity‐related and organizational processes. These inconsistencies may be particularly pronounced among male nurses, whose retention decisions are shaped not only by individual capability but also by the extent to which their professional role is recognized and valued within gendered organizational environments.

Professional self‐concept refers to nurses’ perceptions of themselves as competent, autonomous, and legitimate professionals and represents a core component of professional identity [21]. A strong professional self‐concept has been associated with higher job satisfaction, organizational commitment, and career persistence [22]. For male nurses, professional self‐concept may serve as a critical psychological resource that buffers against gender‐based role conflict and reinforces occupational legitimacy within female‐dominated workplaces [23]. Thus, professional self‐concept may represent a key psychological pathway through which self‐leadership influences retention‐related outcomes.

Organizational commitment reflects emotional attachment, identification with organizational values, and willingness to remain within an organization [24]. High levels of organizational commitment among nurses have been consistently linked to increased intention to stay and reduced turnover [25]. However, male nurses may experience difficulties in developing organizational commitment owing to limited role models, exclusion from informal networks, and perceived inequities in evaluation and promotion [7]. In this respect, organizational commitment may function as a critical organizational‐level mechanism translating individual self‐regulatory capacity into sustained employment decisions.

Taken together, these findings suggest that self‐leadership alone may be insufficient to directly influence retention among male nurses unless it contributes to the development of professional self‐concept and organizational commitment. Conceptually, this implies a mediated framework in which individual self‐regulation enhances identity consolidation and organizational attachment, which in turn shape intention to stay. Understanding how these variables interact is essential for designing effective retention strategies that address both individual capability and organizational context.

Therefore, we aimed to examine the effect of self‐leadership on intention to stay among male nurses and test the mediating roles of professional self‐concept and organizational commitment. By focusing on male nurses, we sought to address a significant gap in the literature and provide evidence‐based implications for nursing management aimed at sustaining a diverse and stable nursing workforce.

## 2. Methods

### 2.1. Design

This study employed a descriptive cross‐sectional survey design to examine the relations among self‐leadership, professional self‐concept, organizational commitment, and intention to stay among male nurses. This study was reported in accordance with the Strengthening the Reporting of Observational Studies in Epidemiology (STROBE) guidelines for cross‐sectional studies.

### 2.2. Participants

Our participants were male nurses currently employed in general or tertiary hospitals in South Korea. The inclusion criteria were as follows: (1) male nurses currently working in clinical practice in a hospital setting, and (2) those with at least 3 months of clinical experience in their current institution to ensure a minimum level of organizational socialization and job exposure. The exclusion criteria included the following: (1) nurses who were not actively engaged in clinical practice (e.g., administrative roles or leave of absence), (2) those with less than 3 months of clinical experience in their current workplace, and (3) responses with incomplete data or inconsistent answering patterns. A total of 210 responses were collected. After excluding 16 questionnaires due to incomplete or inconsistent responses, the final sample consisted of 194 male nurses.

We determined sample size adequacy based on recommendations for mediation analysis using bootstrapping techniques. Previous methodological studies suggest that a sample size of at least 200 is desirable for stable estimation of indirect effects, although smaller samples may be sufficient for regression‐based mediation models [26, 27]. Power analysis using G × Power 3.1 indicated a minimum required sample size of 119 for multiple regression analysis with a medium effect size (*f* ^2^ = 0.15), *α* = 0.05, and power of 0.95. We thus deemed the final sample size adequate for the planned analyses.

### 2.3. Measures

We measured self‐leadership using the Self‐Leadership Scale for Nurses, developed for clinical nurses and consisting of 16 items across four subdomains [28]: collaborative self‐management, physical vitality enhancement, goal‐oriented self‐training, and self‐respect pursuit strategies. Items were rated on a five‐point Likert scale, with higher scores indicating higher levels of self‐leadership. In our study, internal consistency reliability was high (Cronbach’s *α* = 0.91).

We assessed professional self‐concept using the Korean version [29] of the Nurses Self‐Concept Questionnaire originally developed by Cowin [21]. This instrument consists of 36 items across six domains: general self‐concept, caring, staff relations, communication, knowledge, and leadership. Items were rated on an eight‐point Likert scale, with higher scores reflecting a more positive professional self‐concept. The scale demonstrated excellent reliability in our study (Cronbach’s *α* = 0.98).

We measured organizational commitment using the 12‐item Korean version of the Organizational Commitment Scale [24, 30], which covers value, effort, and continuance commitment. Responses were recorded on a five‐point Likert scale, with higher scores indicating stronger organizational commitment. Cronbach’s *α* for this scale was 0.93 in our study.

We measured intention to stay using the Nurses’ Retention Index [5, 31], consisting of six items rated on an eight‐point Likert scale, with two reverse‐coded items. Higher scores indicated stronger intention to remain in the current organization. The reliability of this instrument in our study was high (Cronbach’s *α* = 0.95).

### 2.4. Data Collection Procedure

Data were collected through an online self‐administered questionnaire between July 2024 and August 2025, following approval from the institutional review board. We posted recruitment notices containing a brief explanation of the study purpose and a survey link on online communities and social media platforms commonly used by nurses.

Before accessing the survey, participants read an online information sheet explaining the study objectives, procedures, voluntary nature of participation, confidentiality, and the right to withdraw at any time without penalty. Only participants who provided informed consent electronically were allowed to proceed with the survey. To acknowledge participation, we provided a small mobile gift voucher upon survey completion.

### 2.5. Data Analysis

Data were analyzed using SPSS version 29.0 and the PROCESS macro (Version 4.2). We used descriptive statistics to summarize participants’ demographic characteristics and study variables. We examined differences in self‐leadership, professional self‐concept, organizational commitment, and intention to stay according to general characteristics using independent *t*‐tests and one‐way analysis of variance, with Scheffé tests for post hoc comparisons. We calculated Pearson’s correlation coefficients to examine relations among key variables. We conducted multiple regression analysis to identify predictors of intention to stay while controlling for relevant demographic and job‐related variables. We performed mediation analysis using PROCESS macro (Model 4)—a regression‐based path analytic approach that estimates direct and indirect effects within a mediation framework. Model 4 specifies parallel mediation, allowing the simultaneous examination of multiple mediators. We used bootstrapping with 10,000 resamples to generate bias‐corrected 95% confidence intervals for indirect effects, as bootstrapping does not assume normality of the sampling distribution and is recommended for mediation analysis. Statistical significance was determined by the absence of zero within the confidence intervals [27].

### 2.6. Ethical Considerations

This study was conducted in accordance with the ethical principles of the Declaration of Helsinki. This study was approved by the Institutional Review Board of Hallym University (HIRB‐2024–045). Participation was entirely voluntary, and all participants provided informed consent prior to data collection. Anonymity and confidentiality were ensured by collecting data without personally identifiable information. All data were stored securely and used solely for research purposes. The participants could withdraw from the study at any time without any negative consequences.

## 3. Results

### 3.1. General Characteristics of the Participants

Table 1 summarizes the general characteristics of our participants. The participants were 194 male nurses with a mean age of 28.14 years (SD = 2.06), the majority of whom were younger than 30 years (72.3%) and unmarried (90.7%). Most participants held a bachelor’s degree (86.6%), and the mean length of clinical experience was 2.70 years (SD = 1.54), with nearly 70% having between more than 1 year and less than 4 years of experience. Participants most commonly worked in general wards (42.3%) or intensive care units (24.7%), and the majority were employed on rotating three‐shift schedules (86.1%). Most nurses worked in general hospitals (70.6%), while 29.4% were employed in tertiary hospitals. Overall, 65.5% of participants reported satisfaction with their current workplace, and most indicated no intention to leave their job (74.2%).

**TABLE 1:** General characteristics of the participants (*N* = 194).

Variables	Categories	*n* (%)	Mean ± SD
Age (years)	24–29	141 (72.3)	28.14 ± 2.06
	30–38	53 (27.3)	

Civil status	Single	176 (90.7)	
	Married	18 (9.3)	

Education level	Associate degree	23 (11.9)	
	Bachelor’s degree	168 (86.6)	
	Master’s degree	3 (1.5)	

Clinical experience (years)	≤ 1 year (novice)	18 (9.3)	2.70 ± 1.54
	> 1 to < 4 years (advanced beginner, CN I)	135 (69.6)	
	≥ 4 to ≤ 6.7 years (competent, CN II)	41 (21.1)	

Work unit	General ward	82 (42.3)	
	Intensive care unit	48 (24.7)	
	Operating room/anesthesia	15 (7.7)	
	Emergency department	29 (14.9)	
	Others (e.g., outpatient clinic, endoscopy unit)	20 (10.3)	

Work schedule	Fixed day shift	20 (10.3)	
	Two‐shift rotation	7 (3.6)	
	Three‐shift rotation	167 (86.1)	

Hospital type	General hospital	137 (70.6)	
	Tertiary hospital	57 (29.4)	

Job satisfaction	Dissatisfied	13 (6.7)	
	Neutral	54 (27.8)	
	Satisfied	127 (65.5)	

Turnover intention	No	144 (74.2)	
	Yes	50 (25.8)	

### 3.2. Differences in Intention to Stay According to General Characteristics

Intention to stay did not differ significantly according to age, civil status, education level, work schedule, or hospital type (Table 2). However, clinical experience was significantly associated with intention to stay (*F* = 3.75, *p* = 0.025), with nurses who had more than 1 year of experience reporting higher intention to stay compared with those who had less than 1 year of experience. We also observed significant differences in intention to stay according to work unit (*F* = 5.28, *p* < 0.001), with nurses working in emergency departments and intensive care units reporting higher intention to stay than those working in general wards or other units. In addition, job satisfaction showed a strong association with intention to stay (*F* = 75.61, *p* < 0.001), with satisfied nurses reporting markedly higher intention to stay compared with those who were neutral or dissatisfied. Nurses without turnover intention reported significantly higher intention to stay compared with those who had considered leaving their current workplace (*t* = 11.66, *p* < 0.001).

**TABLE 2:** Differences in intention to stay according to general characteristics (*N* = 194).

Variables	Categories	Intention to stay (*M* ± SD)	t/F (*p*)
Age (years)	24–29	37.24 ± 10.54	−0.47 (0.637)
	30–38	37.96 ± 9.00	

Civil status	Single	37.68 ± 10.02	1.00 (0.159)
	Married	35.17 ± 11.03	

Education level	Associate degree^a^	44.57 ± 3.98	10.40 (< 0.001)^∗∗∗^
	Bachelor’s degree^b^	36.72 ± 10.18	
	Master’s degree^c^	22.33 ± 9.50	

Clinical experience (years)	≤ 1 year^a^	32.00 ± 11.03	3.75 (0.025)^∗^
	> 1 to < 4 years^b^	38.54 ± 9.68	
	≥ 4 to ≤ 6.7 years^c^	36.29 ± 10.46	

Work unit	General ward^a^	39.73 ± 8.98	5.28 (< 0.001)^∗∗∗^
	Intensive care unit^b^	33.19 ± 11.39	
	Operating room/anesthesia^c^	35.67 ± 9.76	
	Emergency department^d^	41.31 ± 6.71	
	Others^e^	34.05 ± 11.56	

Work schedule	Fixed day shift	37.53 ± 9.83	0.49 (0.614)
	Two‐shift rotation	41.14 ± 4.45	
	Three‐shift rotation	37.27 ± 10.33	

*Note:* Values are presented as mean ± standard deviation (M ± SD). Different superscript letters (a, b, c, d, e) indicate statistically significant differences between groups based on Scheffé post hoc tests.

^∗^ *p* < 0.05.

^∗∗∗^ *p* < 0.001.

### 3.3. Relations Among Self‐Leadership, Professional Self‐Concept, Organizational Commitment, and Intention to Stay

Pearson’s correlation analysis revealed significant positive relations among self‐leadership, professional self‐concept, organizational commitment, and intention to stay (Table 3). Self‐leadership was positively correlated with professional self‐concept (*r* = 0.77, *p* < 0.001), organizational commitment (*r* = 0.72, *p* < 0.001), and intention to stay (*r* = 0.63, *p* < 0.001). Professional self‐concept showed strong positive correlations with organizational commitment (*r* = 0.86, *p* < 0.001) and intention to stay (*r* = 0.75, *p* < 0.001). Organizational commitment was also strongly and positively associated with intention to stay (*r* = 0.78, *p* < 0.001), indicating that higher levels of organizational attachment were related to a stronger intention to remain in the current workplace.

**TABLE 3:** Correlations among self‐leadership, professional self‐concept, organizational commitment, and intention to stay (*N* = 194).

Variables	Self‐leadership	Professional self‐concept	Organizational commitment	Intention to stay
Self‐leadership	1			
Professional self‐concept	0.77^∗∗∗^	1		
Organizational commitment	0.72^∗∗∗^	0.86^∗∗∗^	1	
Intention to stay	0.63^∗∗∗^	0.75^∗∗∗^	0.78^∗∗∗^	1

*Note:* Pearson’s correlation coefficients are presented.

^∗^ *p* < 0.05.

^∗∗^ *p* < 0.01.

^∗∗∗^ *p* < 0.001.

### 3.4. Factors Influencing Intention to Stay

Table 4 shows the results of the multiple regression analysis conducted to identify factors influencing intention to stay among our participants. The regression model was statistically significant (*F* = 46.001, *p* < 0.001) and explained 66.9% of the variance in intention to stay (*R* ^2^ = 0.669). Among the independent variables, professional self‐concept (*B* = 0.069, *β* = 0.293, *p* = 0.002) and organizational commitment (*B* = 0.310, *β* = 0.281, *p* = 0.006) were identified as significant positive predictors of intention to stay. In contrast, turnover intention in the current workplace was a significant negative predictor of intention to stay (*B* = −5.241, *β* = −0.225, *p* < 0.001).

**TABLE 4:** Factors influencing intention to stay among male nurses (*N* = 194).

Variables	*B*	SE	*β*	t	*p*	95% CI (LLCI, ULCI)
Constant	9.576	6.045	—	1.584	0.115	−2.352, 21.503
Self‐leadership	0.057	0.079	0.051	0.717	0.475	−0.099, 0.212
Professional self‐concept	0.069	0.022	0.293	3.092	0.002^∗∗^	0.025, 0.114
Organizational commitment	0.310	0.113	0.281	2.754	0.006^∗∗^	0.083, 0.533
Education level	−1.260	1.385	−0.044	−0.910	0.364	−3.992, 1.472
Clinical experience (years)	0.283	0.828	0.015	0.341	0.733	−1.351, 1.917
Work unit	−0.074	0.313	−0.010	−0.238	0.812	−0.691, 0.542
Job satisfaction	1.057	1.183	0.064	0.894	0.372	−1.276, 3.391
Turnover intention	−5.241	1.353	−0.225	−3.873	< 0.001^∗∗∗^	−7.911, −2.571

*Note: B* = unstandardized coefficient; *β* = standardized coefficient.

Abbreviations: CI = confidence interval; LLCI = lower‐limit confidence interval; SE = standard error; ULCI = upper‐limit confidence interval.

^∗^ *p* < 0.05.

^∗∗^ *p* < 0.01.

^∗∗∗^ *p* < 0.001.

Self‐leadership did not show a significant direct effect on intention to stay when controlling for other variables (*B* = 0.057, *β* = 0.051, *p* = 0.475). In addition, educational level, length of clinical experience, work unit, and current job satisfaction were not significantly associated with intention to stay in the adjusted model. Multicollinearity diagnostics indicated no concerns, with all variance inflation factor values being below 10.

### 3.5. Mediating Effects of Professional Self‐Concept and Organizational Commitment

We examined the mediating effects of professional self‐concept and organizational commitment on the relation between self‐leadership and intention to stay using PROCESS macro Model 4 with 10,000 bootstrap samples, controlling for turnover intention in the current workplace. The proposed mediation model specified two independent mediators operating in parallel. Self‐leadership had a significant positive effect on professional self‐concept (*a*_1_ path; *B* = 3.095, SE = 0.228, *t* = 13.58, *p* < 0.001). Self‐leadership also significantly predicted organizational commitment (*a*_2_ path; *B* = 0.569, SE = 0.048, *t* = 11.94, *p* < 0.001).

When entered simultaneously into the model, both professional self‐concept and organizational commitment showed significant positive effects on intention to stay. Professional self‐concept was positively associated with intention to stay (*b*_1_ path; *B* = 0.069, SE = 0.021, *t* = 3.23, *p* = 0.002), as was organizational commitment (*b*_2_ path; *B* = 0.351, SE = 0.103, *t* = 3.42, *p* = 0.001). In contrast, the direct effect of self‐leadership on intention to stay was not statistically significant when the mediators were included (c′ path; *B* = 0.083, SE = 0.075, *t* = 1.11, *p* = 0.269) (Table 5).

**TABLE 5:** Mediating effects of professional self‐concept and organizational commitment on the relation between self‐leadership and intention to stay (*N* = 194).

Pathway	*B*	SE	t	*p*	95% CI (LLCI, ULCI)
*Model 1. Professional self-concept (* *M*_1_ *)*					
Self‐leadership ⟶ professional self‐concept (*a*_1_)	3.095	0.228	13.575	< 0.001^∗∗∗^	2.646, 3.545

*Model 2. Organizational commitment (* *M*_2_ *)*					
Self‐leadership ⟶ organizational commitment (*a*_2_)	0.569	0.048	11.942	< 0.001^∗∗∗^	0.475, 0.662

*Model 3. Intention to stay (Y)*					
Self‐leadership ⟶ intention to stay (c′)	0.083	0.075	1.105	0.269	−0.064, 0.230
Professional self‐concept ⟶ intention to stay (*b*_1_)	0.069	0.021	3.230	0.002^∗∗^	0.027, 0.113
Organizational commitment ⟶ intention to stay (*b*_2_)	0.351	0.103	3.426	0.001^∗∗^	0.147, 0.553
Total effect (c)	0.496	0.059	8.377	< 0.001^∗∗∗^	0.379, 0.613

*Note:* Bootstrap resampling = 10,000. *B* = unstandardized coefficient.

Abbreviations: CI = confidence interval; LLCI = lower‐limit confidence interval; SE = standard error; ULCI = upper‐limit confidence interval.

^∗^ *p* < 0.05.

^∗∗^ *p* < 0.01.

^∗∗∗^ *p* < 0.001.

**FIGURE 1:**
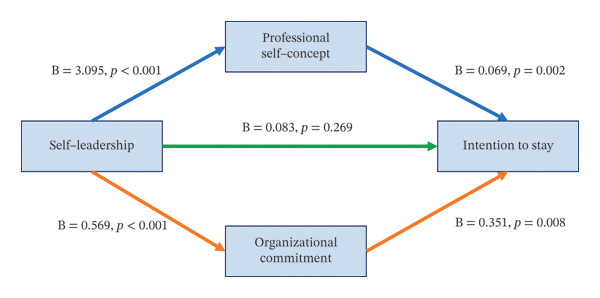
Independent multiple mediation model of the effect of self‐leadership on intention to stay in the job.

The total effect of self‐leadership on intention to stay was statistically significant (c path; *B* = 0.496, SE = 0.059, *t* = 8.38, *p* < 0.001). Bootstrapping analysis confirmed significant indirect effects through professional self‐concept (*B* = 0.214, 95% CI [0.028, 0.396]) and organizational commitment (*B* = 0.199, 95% CI [0.058, 0.351]). The total indirect effect of self‐leadership on intention to stay was also significant (*B* = 0.413, 95% CI [0.248, 0.576]). The difference between the two indirect effects was not statistically significant (Figure 1).

## 4. Discussion

This study examined the relationship between self‐leadership and intention to stay among male nurses and investigated the mediating roles of professional self‐concept and organizational commitment. The findings provide a theoretically nuanced understanding of how individual regulatory capacities translate into retention‐related outcomes within a gendered professional context.

First, although self‐leadership was positively correlated with intention to stay, its direct effect became nonsignificant after professional self‐concept and organizational commitment were included in the model. This pattern indicates that self‐leadership does not independently determine retention decisions but operates indirectly through psychological and organizational mechanisms. This finding helps reconcile inconsistencies in prior research reporting mixed results regarding the direct association between self‐leadership and turnover‐related outcomes [19, 20]. Rather than functioning as a proximal determinant of retention, self‐leadership appears to serve as an upstream individual resource that shapes how nurses construct professional identity and organizational attachment.

The absence of a direct pathway is theoretically consistent with perspectives suggesting that individual leadership capabilities must be embedded within identity and relational processes to influence long‐term organizational outcomes [16, 17]. From a social identity perspective, retention decisions are shaped not solely by competence but by the extent to which professional identity is socially validated within the organizational context. For male nurses working in female‐dominated professional environments, self‐regulatory capacity may enhance performance and resilience; however, these internal resources alone may be insufficient to cultivate belongingness and sustained career commitment. Belonging and organizational embeddedness are influenced by relational validation, role recognition, and inclusive workplace norms. In contexts where gender‐based role incongruity or minority status persists, retention is likely contingent upon whether professional identity is affirmed and organizational attachment is reinforced. Thus, individual regulatory capacity must interact with supportive organizational structures to translate competence into sustainable career engagement.

Second, professional self‐concept emerged as a significant mediator between self‐leadership and intention to stay. Self‐leadership was strongly associated with professional self‐concept, which in turn predicted intention to stay. This underscores the centrality of professional identity in shaping retention decisions. Professional self‐concept reflects nurses’ perceptions of competence, legitimacy, and value within their professional role [21]. For male nurses, who may encounter role incongruity and gender‐based stereotypes, a well‐developed professional self‐concept may be specifically essential in reinforcing occupational legitimacy and long‐term career commitment [7, 23]. Consistent with previous findings linking professional self‐concept to job satisfaction, organizational commitment, and career persistence [22, 32], our results extend prior research by demonstrating that professional identity functions as a key psychological mechanism through which self‐leadership contributes to retention.

Third, organizational commitment also functioned as a significant mediator. Self‐leadership was positively associated with organizational commitment, which in turn was a strong predictor of intention to stay. This finding aligns with extensive literature identifying organizational commitment as a robust determinant of nurse retention [24, 25]. Individuals who demonstrate higher levels of self‐regulation and proactive goal orientation may be more likely to develop affective attachment to their organizations, as self‐leadership enhances perceived autonomy, ownership, and alignment with organizational goals [16, 17]. Within nursing contexts, organizational commitment has consistently been associated with reduced turnover intention and increased career persistence, particularly when fairness, recognition, and supportive leadership are present [1, 25]. However, male nurses often report limited role models, exclusion from informal networks, and inequities in advancement opportunities [12, 14], all of which may constrain the development of organizational commitment. Our findings suggest that when self‐leadership fosters stronger organizational attachment, it becomes a meaningful pathway to retention.

Importantly, the total indirect effect of self‐leadership on intention to stay was significant, whereas the direct effect became nonsignificant after inclusion of the mediators, indicating a full mediation structure. In other words, the association between self‐leadership and intention to stay was entirely transmitted through professional self‐concept and organizational commitment rather than operating through a direct pathway. This reinforces the view that retention is a multilevel process shaped by interactions among individual regulatory capacity, professional identity formation, and organizational attachment.

Contextual factors further illuminate retention dynamics. Nurses with less than 1 year of clinical experience reported lower intention to stay in univariate analysis, consistent with literature documenting elevated turnover risk and transitional challenges during early professional socialization. Reality shock, workload stress, and adjustment difficulties may weaken early organizational attachment. Although clinical experience was not significant in the adjusted model, this pattern highlights the importance of structured orientation and mentorship during the first year of practice. Additionally, differences across work units were observed, with nurses in emergency departments and intensive care units reporting higher intention to stay than those in general wards. This may reflect greater role specialization, professional autonomy, and skill development in high‐acuity settings. Nevertheless, unit differences may partially reflect variations in clinical experience or selective placement processes. Although experience was statistically controlled, future research should examine the interaction between work unit, career stage, and retention trajectories.

The practical implications are particularly relevant for nursing management. Leadership development initiatives that strengthen self‐regulatory strategies—such as goal‐setting, constructive cognitive strategies, self‐observation, and intrinsic motivation—may be insufficient if implemented in isolation. Retention strategies should move beyond individual capability enhancement toward integrated, multilevel interventions. Organizational efforts that support professional identity formation and foster inclusive workplace cultures are essential. Mentorship programs, equitable role allocation, recognition of professional contributions, and transparent career development pathways may collectively strengthen professional self‐concept and organizational commitment among male nurses [8, 25].

Several limitations should be acknowledged. The cross‐sectional design precludes causal inference and limits conclusions regarding temporal ordering among variables. Although mediation was tested statistically, longitudinal designs are necessary to confirm directional pathways. Data were collected via self‐report, introducing potential response bias. The sample consisted primarily of relatively young male nurses recruited through online communities, which may have introduced selection bias and limits generalizability to older or less digitally engaged populations. Additionally, the single national context may restrict international applicability. Future research should employ longitudinal and comparative designs, including both male and female nurses, to examine gender‐specific mechanisms and contextual influences on retention.

Despite these limitations, this study advances the understanding of the psychological and organizational mechanisms linking self‐leadership to intention to stay among male nurses. By demonstrating the mediating roles of professional self‐concept and organizational commitment, the findings provide a theoretically grounded and practically relevant framework for designing retention strategies in gendered professional environments.

## 5. Conclusion

This study demonstrated that self‐leadership influences male nurses’ intention to stay indirectly through professional self‐concept and organizational commitment rather than through a direct pathway. These findings highlight that individual leadership capacity alone is insufficient to sustain retention in gendered nursing environments. Self‐leadership becomes meaningful for retention when it strengthens professional identity and fosters organizational attachment. For nursing management, this underscores the importance of integrating leadership development initiatives with organizational strategies that promote inclusive cultures, professional recognition, and commitment‐building practices. By addressing both individual and organizational dimensions, healthcare organizations can develop more effective retention strategies and support the long‐term sustainability of a diverse nursing workforce.

## Author Contributions

Donghyeok Lee conceptualized and designed the study, collected the data, and drafted the initial manuscript. Mihwa Shim contributed to data interpretation and provided critical feedback during manuscript revision. Jungmin Lee, as the corresponding author, supervised the overall research process, guided methodological decisions, contributed to data analysis and interpretation, and critically revised and finalized the manuscript.

## Funding

This work was supported by the Hallym University Research Fund, 2025 (HRF‐202509–002).

## Disclosure

All authors read and approved the final manuscript and agree to be accountable for all aspects of the work.

## Ethics Statement

This study was approved by the Institutional Review Board of Hallym University (HIRB‐2024–045). All participants were informed of the study purpose, procedures, potential risks, and their rights as research participants. Written informed consent was obtained prior to participation. Participation was voluntary, and anonymity and confidentiality were assured. No identifiable personal information was collected, and all data were used solely for research purposes.

## Consent

Please see the Ethics Statement.

## Conflicts of Interest

The authors declare no conflicts of interest.

## Data Availability

The datasets generated and analyzed during the current study are available from the corresponding author upon reasonable request.
